# A random walk approach to estimate the confinement of α-particle emitters in nanoparticles for targeted radionuclide therapy

**DOI:** 10.1186/s41181-018-0042-3

**Published:** 2018-05-30

**Authors:** Uwe Holzwarth, Isaac Ojea Jimenez, Luigi Calzolai

**Affiliations:** European Commission, Joint Research Centre, Via Enrico Fermi 2749, 21027 Ispra, VA Italy

**Keywords:** Targeted radionuclide therapy, α-particle emitters, ^225^Ac, ^224^Ra, ^223^Ra, Recoil energy, Confinement of daughter radionuclides in nanoparticles, Nanocarriers, Nanomedicine

## Abstract

**Background:**

Targeted radionuclide therapy is a highly efficient but still underused treatment modality for various types of cancers that uses so far mainly readily available β-emitting radionuclides. By using α-particle emitters several shortcomings due to hypoxia, cell proliferation and in the selected treatment of small volumes such as micrometastasis could be overcome. To enable efficient targeting longer-lived α-particle emitters are required. These are the starting point of decay chains emitting several α-particles delivering extremely high radiation doses into small treatment volumes. However, as a consequence of the α-decay the daughter nuclides receive high recoil energies that cannot be managed by chemical radiolabelling techniques. By safe encapsulation of all α-emitters in the decay chain in properly sized nanocarriers their release may be avoided.

**Results:**

The encapsulation of small core nanoparticles loaded with the radionuclide in a shell structure that safely confines the recoiling daughter nuclides promises good tumour targeting, penetration and uptake, provided these nanostructures can be kept small enough. A model for spherical nanoparticles is proposed that allows an estimate of the fraction of recoiling α-particle emitters that may escape from the nanoparticles as a function of their size. The model treats the recoil ranges of the daughter nuclides as approximately equidistant steps with arbitrary orientation in a three-dimensional random walk model.

**Conclusions:**

The presented model allows an estimate of the fraction of α-particles that are emitted from outside the nanoparticle when its size is reduced below the radius that guarantees complete confinement of all radioactive daughter nuclides. Smaller nanoparticle size with reduced retention of daughter radionuclides might be tolerated when the effects can be compensated by fast internalisation of the nanoparticles by the target cells.

## Introduction

Targeted radionuclide therapy in cancer treatment is a rapidly evolving field that achieved major success with the U.S. Food and Drug Administration's (FDA) approval of two antibodies targeting CD20 radiolabelled with β-emitters in 2002 (^90^Y-labeled ibritumomab tiuxetan, commercialized as Zevalin) and 2003 (^131^I-labeled tositumomab, commercialized as Bexxar) for therapy of B-cell non-Hodgkin's lymphoma (Goldsmith, 2010; Bodet-Milin et al., 2013).

Targeted radionuclide therapy has the potential to treat cancer by delivering locally therapeutic radiation doses even in disseminated disease where beam radiotherapies are not applicable. Its application field is steadily broadening by the identification of new specific targets on membranes of cancer cells and new vectors targeting them. The state of the art and the vast amount of experience gained with radiolabeled monoclonal antibodies, antibody fragments, peptides, organ-specific proteins and other small molecules has been compiled by Baum (2014). This comprehensive work underlines the so far dominating role of β-emitters such as ^131^I, ^90^Y, ^188^Re, ^177^Lu.

The range of the emitted β-radiation covers distances from fractions of mm to several mm depending on the used radionuclide. Since this range is equivalent to hundreds of cell diameters, the so-called "cross-fire effect" may destroy cancer cells in the neighborhood of those having successfully been targeted, thus, overcoming problems of tumour cell heterogeneity and drug penetration in tumors (Haberkron et al., 2017; Aghevlian et al., 2017; Elgqvist et al., 2014; Kassis, 2008). However, due to the limited linear energy transfer (LET) of β-radiation a cell must be hit by many thousands of β-particles before it is successfully killed and high activities have to be applied (Elgqvist et al. 2014, Kassis 2008). Therefore, in the case of micrometastatic or residual disease, the largest part of the β-radiation is delivered to healthy tissue even though targeting may be successful.

The use of α-particles emitters solves this problem, since α-particles have a very short range in tissue corresponding to a few cell diameters only, on which they deposit their whole kinetic energy of several MeV (Kassis, 2008). Thus, their LET is two orders of magnitude higher than those of β-particles, and α-particles cause a very high ionisation density around the particle trajectory efficiently inducing DNA double strand breaks, resulting in high cell toxicity and cell death predominantly by apoptosis (Kassis, 2008). In comparison, the toxicity of β-particles is mediated by the creation of free radicals in oxygen species that indirectly damage DNA. Therefore, DNA damage caused by β-particles can be more efficiently repaired by cells than that caused by α-particles, where 1 – 5 hits may be sufficient to cause cell death (Elgqvist et al., 2014; Kim and Brechbiel, 2012; Kassis, 2008). These differences imply that α-emitters can deliver therapeutic radiation doses to small volumes at high dose rate and their toxicity is independent of cell proliferation and tissue oxygenation. This helps to overcome hypoxia as limiting factor for the efficiency of radiotherapy, it does not require dose fractionation and breaks resistance to chemotherapy and therapy with low-LET radiation (Haberkorn et al., 2017; Seidl, 2014; Elgqvist et al., 2014). The locally very high toxicity of α-emitters may also (partially) compensate for lower tumour uptake of radiolabelled vectors (Allen, 2017). However, concomitantly this high toxicity requires vectors with especially high affinity, high specificity, good *in vivo* stability and fast uptake by target cells since circulation times as long as or even longer than the physical half-live of the radionuclide can cause unspecific off-target irradiation and unintentional toxicity (Aghevlian et al., 2017; Seidl, 2014). The majority of pre-clinical and clinical trials have demonstrated that α-emitters such as ^225^Ac, ^211^At, ^212^Bi, ^213^Bi, ^212^Pb, ^223^Ra and ^227^Th are ideal for the treatment of micro-metastatic disseminated disease and of smaller tumor burdens, e.g. of residual disease after surgery (Aghevlian et al., 2017; Allen, 2013; Kim and Brechbiel, 2012).

These promising pre-clinical results have been confirmed by the clinical application in the treatment of serious diseases such as leukaemia (e.g., Rosenblat et al., 2010), lymphoma (Heeger et al., 2003), recurrent ovarian cancer (Meredith et al., 2014; Andersson et al., 2009), metastatic melanoma (Allen et al., 2011), neuroendocrine tumours (Kratochwil et al. 2014), glioma (Zalutsky et al., 2008; Cordier et al., 2010; Cordier et al., 2016) and metastatic castration-resistant prostate cancer even in disease refractory to β-treatment (Kratochwil et al. 2016). Unfortunately, a broader use of α-particle emitting radionuclides is hampered by the following issues: (i) With very few exceptions, α-particle emitting radionuclides are not readily available and are therefore still expensive. This issue has been reviewed by various authors (Seidl, 2014; Elgqvist et al., 2014), and technologies have been developed that could enable practically unlimited supply if a substantial commercial demand is once established (Apostolidis et al., 2005; Weidner et al., 2012). (ii) Adequate α-particle emitting radionuclides are either too short-lived for most targeting approaches or they are sufficiently long-lived but exhibit a complex α-particle-emission cascade (Elgqvist et al., 2014; Couturier et al., 2005). In the case of α-emitters with a short half-life of one hour or less, the first successful targeting using monoclonal antibodies (mAbs) was restricted to leukemic cells that could be reached by mAbs within minutes after intravenous injection (Aghevlian et al., 2017; Allen, 2013) and to locoregional applications. (iii) Longer-lived α-emitters exhibit complex decay chains in which the daughter radionuclides receive high recoil energies of typically about 100 keV when the α-particle is emitted. This energy is about four orders of magnitude higher than any chemical binding energy, which means that the daughter radionuclide cannot be hold by a chemical bond. Therefore, when using bifunctional ligands, the daughter nuclide will be released, may reach the blood stream, will be displaced and cause off-target toxicity (de Kruijff et al., 2015; Jaggi et al., 2005).

One application of a long-lived mother radionuclide in an α-particle cascade that is not affected by these limitations is the intravenous administration of ^223^RaCl_2_ in patients to treat bone metastases from prostate cancer (Pöppel et al., 2016; Kluetz et al., 2014). Due to its natural affinity to bone tissue the accumulation of radium is sufficiently fast and persistent that all daughter nuclides ^219^Rn, ^215^Po, ^211^Pb, ^211^Bi und ^207^Tl can contribute to the therapeutic effect. The aqueous ^223^RaCl_2_ solution is licensed by the European Medicines Agency for treatment of adults with castration resistant prostate carcinoma with symptomatic bone metastasis without known visceral metastasis, and in May 2013 it was approved by the U.S. FDA for the treatment of bone metastases from prostate cancer as the first α-emitting radiopharmaceutical for clinical use (Kluetz et al., 2014). Aqueous ionic ^223^RaCl_2_ is supplied as a product ready for use (Xofigo®)^1^with an activity of 6.6 MBq at the reference date and a specific activity of 1.9 MBq/ng. The treatment scheme comprises 6 injections every 4 weeks with a dose of 55 kBq/kg BW (body weight) each (Pöppel et al., 2016).

In cases where no natural affinity of the α-emitting mother radionuclide to the target tissue can be exploited there are essentially three strategies to directly use the long-lived mother radionuclide in the decay chain for therapy (de Kruijff et al., 2015). (i) Local application: The compound must be applied locoregionally in a compartment with no or sufficiently slow exchange with the surrounding in order to ensure that no daughter radionuclides may infiltrate blood circulation until the stable nuclide at the end of the decay chain is formed. (ii) Internalisation: If the vectors with the long-lived α-emitters are internalised by the target cells, the cell volume is usually large enough to keep all recoiling daughter radionuclides inside the target cells (McDevitt et al., 2001). This explains why the use of ^225^Ac bound to a targeting molecule by complexation has shown therapeutic efficacy without significant toxicity (Kratochwil et al., 2016). Carrier-mediated internalization was efficacious *in vitro* and *in vivo* for the targeted cells and limited the toxicity to non-target cells (Zhu et al., 2016). (iii) Encapsulation: The third option, discussed here, is to encapsulate the mother radionuclide in a nanoparticle that is big enough to physically confine all recoils in the decay chain in its structure. Such a nanoparticle requires a suitable size and structure and should allow an adequate surface functionalisation to enable systemic administration and efficient tissue targeting.

With the use of longer-lived α-emitters, much more time is available for efficient targeting and the high specificity of mAbs can be exploited, thus covering a much larger variety of tumours. Additionally, with the longer half-life of the parental radionuclides sufficient time would be available to execute the synthesis, labelling and quality control in central radiopharmacies and distribute the products from there to the applying hospitals (Kim and Brechbiel, 2012). Finally, much more economic use could be made of the precious radionuclides (Allen, 2017).

## Review

### Experimental approaches to *in vivo* α-particle nanogenerators

In the exploration of nanoparticles as drug carriers several approaches have been presented in literature to load nanoparticles with α-emitting radionuclides in order to dissipate the recoil energies of the daughter radionuclides in the nanoparticles and to avoid release. This concept of "*in vivo* α-particle generators" was later termed "nanogenerators" by McDevitt et al. (2001). An overview on the experimental results that have so far been reported is presented in Table 1.

**Table 1:** Compilation of encapsulation experiments reported in literature

Nanoparticle type and size	label	labelling yield/label leaching	Retention/release of daughter nuclides	remarks	reference
Zwitterionic pegylated phosphatydylcholine cholesterol liposomes 200 / 400 / 650 nm	^225^Ac	^225^Ac retention > 88% for zwitterionic liposomes after 30d	^213^Bi retention ≈ 12% for 650 nm size liposomes after 2 days and ≈4% after 30 days	Retention values for ^225^Ac and ^213^Bi lower for cationic liposomes	Sofou et al., 2004
liposomes	^225^Ac	Yield (73±9)% / retention up to (81±7)% achievable		Funtionalised liposomes maintain targeting efficacy after ^225^Ac loading	Chang et al., 2008
Polymersomes 100 / 200 /400 /800 nm filtered fractions	^225^Ac	(67±0.8)% in 30 min leaching - 200 nm: 2% after 8d 7% after 28d	highest retention after 24h 800 nm: ^221^Fr (69±1.5)% ^213^Bi (53±4)% 100 nm: ^221^Fr (37±4)% ^213^Bi (22±1)%	Polymerosomes can be internalized by target cells	Wang et al., 2014
[^225^Ac]InPO_4_ nanoparticles Inside polymersomes 100 / 200 /400 /800 nm filtered fractions	^225^Ac	Retention of ^225^Ac in polymersomes containing [^225^Ac]InPO_4_ nanoparticles (92±3)%	retention after 24h 100 nm: ^221^Fr ≈ 57% ^213^Bi ≈ 40%	Amorphous [^225^Ac]InPO_4_ nanoparticles (≈ 20 nm) were created inside the polymersomes; works well for polymersomes < 400 nm	De Kruijff et al., 2017
Hydroxylapatite XRD: 15 nm	^223^Ra	99% yield achievable /Release after 24 h in saline 0.7% (surface absorption) 0.8% (in synthesis labelling)		Labelling during synthesis and after synthesis (surfacesorption) show both very low leaching – re-absorption on surface?	Kozempel et al., 2015
Hydroxylapatite TEM: nanoplates ≤ 100nm x ≤ 500nm x 0.8…2.4 nm	^223^Ra	Labelling yield (97±1)% in 20 h /6% - 15% released within 24h depending on loading strategy		After surface sorption: 8% loading during synthesis: 15% + annealing 900^o^C, 3h: 6% release during 24h	Vasiliev et al., 2016
Nanozeolite XRD 43 nm SEM 50-170 nm DLS 40-120 nm	^224^Ra ^225^Ra	Labelling yield >99.9% leaching < 0.5% (4d)	Release 1d after incubation ^224^Ra: ^212^Pb (2.6±1.5)% ^208^Tl (7.9±0.9)% ^212^Pb (6.2±0.5)% Release 1d after incubation ^225^Ra: ^225^Ac (2.3±1.9)% ^221^Fr(2.7±0.2)% ^213^Bi(7.4±0.8)%	daughter release data reported in human blood serum (data available also for other media)	Piotrowska et al., 2013
Functionalised Nanozeolite-silane-PEG-SP(5-11)TEM: 60 nm	^223^Ra	labelling yield > 99.9% leaching < 0.5%	released daughter nuclides ^211^Pb and ^211^Bi increasing from ≈2% (1d) to ≈5% (6d), each	High receptor affinity preserved; intravenous application not possible	Piotrowska et al., 2017
Fe3O4 SPIONS TEM: 4…26 nm DLS: 284 nm	^223^Ra	Yield in 0.9% NaCl ≤ 50% , in PBS 85-99% within 1h Leaching in bovine serum and plasma 1.5% (11.4d) to 2% (22.8d)		Labelling by surface complexation suggested	Mokhodoeva et al., 2016
{La 0.5 Gd 0.5}PO4 core (2*.*9±0*.*7) nm +4 shells GdPO_4_ + external Au shell 27 nm (with Au shell) (22.4±7.7)nm	^225^Ac	76% after 4 days of core synthesis ^225^Ac retention > 99.9% after 3 weeks	^221^Fr retention ≈90% after 3 weeks	Ac will co-crystallize into a lanthanide phosphate crystal Gd allows separation of ^225^Ac-labelled NPs from co-produced Au-NPs	McLaughlin et al., 2013

The type and size of nanocarriers is presented, the loaded radionuclide and the achievable loading yield and the retention of the mother nuclide. As far as reported the retention of the daughter nuclides are presented

Due to their frequent use as drug carriers in nanomedicine (Bozzuto and Molinari, 2015; Puri et al., 2009; Immordino et al., 2006) liposomes have been investigated both theoretically and experimentally to retain recoils in the decay chains of ^225^Ac (Chang et al., 2008; Sofou et al., 2007; Sofou et al., 2004; Henriksen et al., 2004) and of ^223^Ra (Jonasdottir et al., 2006; Henriksen et al., 2004). The potential advantage of liposomes as nanocarriers was emphasized by Sofou et al. (2004) who determined a radioactive load of 10 to 40 ^225^Ac-atoms per liposome. However, not even the mother radionuclide^225^Ac could completely be retained, and ^213^Bi-retention was as low as 10%, even for the largest liposomes (650 nm) used, and much lower than the expected 50% (Sofou et al., 2004). Chang et al. (2008) used multi-layered liposomes with diameters of up to 750 nm and could achieve approximately 98% of ^225^Ac-retention, while the retention of ^213^Bi as the last α-emitting daughter was still as low as 20%. Based on this poor performance in combination with the large carrier size, liposomes do not seem to be the first choice for systemic therapeutic approaches. However, *in vitro* experiments performed by Zhu et al. (2016) with lipid vessels of about 100 nm size, loaded with ^225^Ac and targeted with the antibody J591 against PSMA on human endothelial cells (HUVEC), could achieve 3 times higher cell killing efficacy compared with the same amount of ^225^Ac activity directly labelled to J591. The authors explained this result by a pronounced perinuclear localisation of the carrier vessels after their internalisation in contrast to directly ^225^Ac-labelled antibodies (Zhu et al., 2016). Hence, for systemic applications such as targeting capillary endothelial cells in *tumour antivascular targeted α-therapy*, where the application of α-particle emitters showed also therapeutic effect in bulky tumours (Allen, 2013; Chan et al., 2016), also the easy and rapid internalisation of the carrier and the location of the α-emitters inside the target cells have to be considered, and many parameters need to be balanced in order to optimise therapeutic effects.

Thijssen et al. (2012) investigated the feasibility to substitute liposomes by polymersomes to achieve higher recoil retentions in smaller carriers. The authors concluded that double-layered polymersomes of 300 – 400 nm size loaded with ^225^Ac could fully retain the first daughter ^221^Fr and nearly 50% of the last α-emitter ^213^Bi. Retention of 80% of the ^213^Bi would again require larger structures of about 800 nm in diameter (Thijssen et al., 2012). In a further experimentally refined study, the same group (Wang et al., 2014) encapsulated ^225^Ac in multilayered polymersomes and varied the diameter of the inner and outer layers as well as the thickness of the membranes. The authors found that double layered polymersomes with external diameters larger than 800 nm can retain ^221^Fr recoils to (69 ± 1.5)% and ^213^Bi to (53 ± 4)%, as determined 24h after loading. This was less than predicted by the sophisticated model developed by Thijssen et al. (2012) that considers also diffusional displacements of the radionuclides inside the carriers. However, an accompanying *in vitro* study with HeLa cells showed that polymersomes can be internalised by endocytosis and are actively transported close to the cell nucleus within less than 1 hour after exposure (Wang et al., 2014). While it is unlikely to achieve adequate tumour targeting with polymersomes of such dimensions due to limited extravasation and tumour penetration and because they will be cleared from the blood stream more efficiently than smaller ones, it appears promising to investigate whether lower recoil retention by smaller constructs might be compensated by faster targeting and fast internalisation. In any case, compared to liposomes that exhibit a fixed membrane thickness of 3-4 nm, the membrane thickness of polymersomes can be controllably varied in the range of 3-200 nm, which renders polymersomes more stable *in vivo*, less permeable and offers possibilities to further tune their properties (Thijssen et al., 2012).

In order to ensure sufficient retention of all recoils within nanoparticles, materials with a higher density than that of liposomes or polymersomes will be required. Additionally the loading with the α-particle emitter must be feasible with high yield. Kozempel et al. (2015) and Vasiliev et al. (2016) investigated the possibility to load ^225^Ac onto hydroxyapatite nanoparticles by adsorption or to incorporate ^225^Ac during nanoparticle synthesis. Hydroxyapatite was selected due to its known biocompatibility. While Kozempel et al. (2015) could achieve an ^225^Ac-retention of better than 99% during 24h with primary nanoparticles of 15 nm size, Vasiliev et al. (2016) had less favourable results with their larger nanoplatelets which retained in the best case 94% of the ^225^Ac. Neither Kozempel et al. (2015) nor Vasiliev et al. (2016) provided data on the release or retention of daughter nuclides.

So far most clinical studies were performed with ^213^Bi obtained from ^225^Ac/^213^Bi generators that can be considered the "work horse for ongoing research" (Allen 2013). This holds also for attempts to use the mother radionuclide ^225^Ac directly to improve tumour infusion that usually requires more time than that available with the short-lived ^213^Bi.

An approach to use the more readily available Ra radionuclides (^223^Ra, ^224^Ra, ^225^Ra) for therapy that form only very weak complexes was developed by the group of Bilewicz using nanometer-sized zeolites (Piotrowska et al., 2013; Piotrowska et al., 2017). Zeolites are biocompatible crystalline aluminosilicates with tetrahedral structures that offer an open framework of molecular dimensions in which metal cations (e.g. Na^+^, K^+^, Ca^2+^) are present in order to render the structure electrically neutral (Piotrowska et al., 2013). The Na A-type zeolite was chosen because it provides the highest selectivity for Ra^2+^ ions and exhibits a window size of 0.42 nm that matches well with the ionic radii of Ra^2+^ and others such as Fr^+^ that appear in the decay chain (Piotrowska et al., 2013). These nanozeolites can efficiently be loaded with radioactive Ra^2+^ ions by ion exchange, and ^223^Ra^2+^, ^224^Ra^2+^ and ^225^Ra^2+^ are well retained in the nanozeolites when suspended in various biological fluids and human serum (Piotrowska et al., 2013). The release of daughter nuclides in the decay chain was quantified in various media. In Table 1 the data obtained in human serum are reported, which appear to be the most relevant for the envisaged medical application.

Motivated by encouraging results of the treatment of glioma patients (WHO grades II–IV) with intratumourally injected ^213^Bi–DOTA–SP, where tumour targeting is achieved by Substance P (SP) (Cordier et al., 2010 and 2016), Piotrowska et al. (2017) functionalised their nanozeolite carriers with Substance P using silane-PEG-SP molecules and could place on average 18.000 molecules on the surface of a 50 – 80 nm diameter zeolite nanoparticle (Piotrowska et al., 2017). The resulting functionalised nanoparticles had a typical hydrodynamic diameter of 160 nm and a ζ-potential between -20 mV and -30 mV with no tendency to aggregate in aqueous suspensions during an observation period of 11 days (Piotrowska et al., 2017). In human serum the leakage of ^223^Ra from the bioconjugate was below 0.5%, and the release of ^211^Pb and ^211^Bi was in the range of 2% to 5%, which corresponds to 90% to 95% retention of the decay products (Piotrowska et al., 2017). This retention was higher than expected for the size of the nanozeolites and was tentatively explained by re-absorption of especially ^219^Rn and ^211^Pb on the zeolite due to the high affinity for these elements (Piotrowska et al., 2017). If this explanation is valid, it is however uncertain whether it can be transferred from *in vitro* conditions to *in vivo* models where blood flow may rapidly dislocate the decay products from the surface of the zeolite nanoparticles, which might reduce the re-adsorption probability. A second tentative explanation given by Piotrowska et al. (2017) that a part of the recoil energy might be transferred to the entire nanoparticle is more unlikely. From momentum conservation it follows that the kinetic energy transferred to the whole nanoparticle is of the order of only one eV, which is a negligible fraction of the kinetic energy of the α-particle.

Up to here, investigations using liposomes and polymersomes with a density close to 1 g⋅cm^-3^, and with hydroxyapatite and zeolite having a density of about 2.5 g⋅cm^-3^, have been reported. In these cases the radioactive payload of the nanoparticles is either located on the nanoparticle's porous surface or homogeneously distributed in the nanoparticle volume depending on the loading or synthesis conditions. Therefore, recoiling daughter nuclides produced by an α-decay on or close to the nanoparticle surface will always have a chance to escape the nanoparticle. McLaughlin et al. (2013) have presented a strategy localising the radionuclides in the centre of the nanoparticles. Provided that the particles are big enough no recoil should be released whatever its emission direction is. These authors start from a small [^225^Ac]{La_0.5_Gd_0.5_}PO_4_ core-nanoparticle with a diameter of about 3 nm in which the radionuclide ^225^Ac is co-crystalized with LaPO_4_ and GdPO_4_. The washed core nanoparticles are then subjected to further growth steps in which shells of LaPO_4_ and/or GdPO_4_ are produced. In order to improve the biocompatibility of these constructs they may be covered with a gold shell. Depending on the total thickness of the shells and the type of materials used, it is possible to confine all recoiling daughter nuclides inside the nanoparticles. Nanoparticles with a total diameter of 23 nm were reported to retain^225^Ac quantitatively and about 90% of ^221^Fr over a period of 30 days (McLaughlin et al., 2013). Although retention data for ^217^At and ^213^Bi were not presented, by increasing the thickness of the shells it can be expected to achieve a nearly complete retention even for the last α-particle emitting daughter nuclide.

A sophisticated combination of McLaughlin's approach (McLaughlin et al., 2013) to localise ^225^Ac inside a nanocarrier with the use of polymersomes (Thijssen et al., 2012; Wang et al., 2014) has been put forward by de Kruijff et al. (2017). These authors succeeded to synthesize amorphous [^225^Ac]InPO_4_ nanoparticles by co-precipitation inside polymersomes. For polymersomes with a diameter of 100 nm the retention of ^221^Fr and ^213^Bi could be improved from 37% to 57% and from 22% to 40%, respectively. For polymersomes larger than 400 nm the retention fractions fell below the expectation because [^225^Ac]InPO_4_ nanoparticles could no longer be created reliably inside the carriers (de Kruijff et al., 2017).

## A systematic approach to nanoparticles as α-particle nano-generators

### Requirements for recoil confinement derived from α-particle decay schemes

**Fig. 1:**
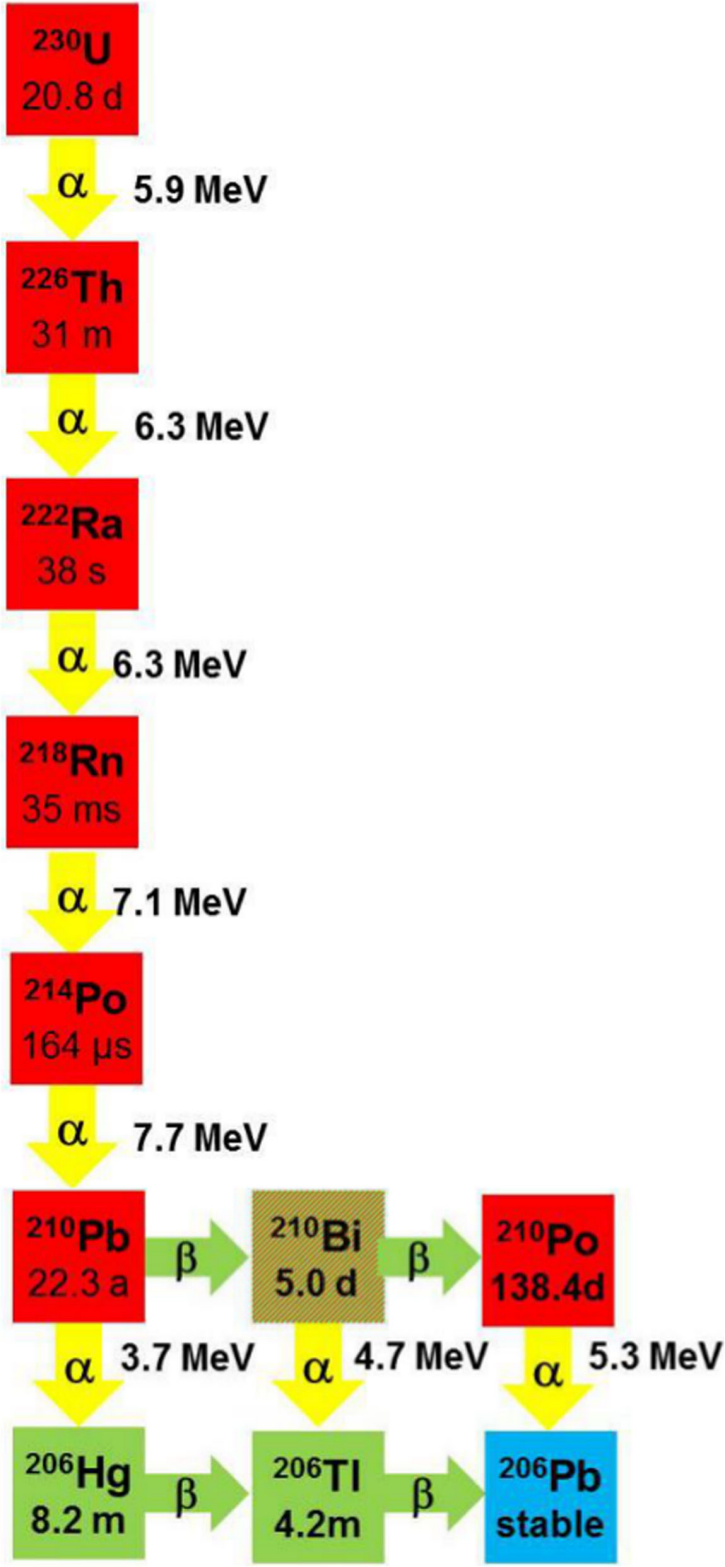
^230^U decay scheme (Data retrieved from J. Magill, G. Pfennig, J. Galy (2006), Karlsruher Nuklidkarte, 7th ed.; Haberbeck GmbH, Germany)

**Fig. 2:**
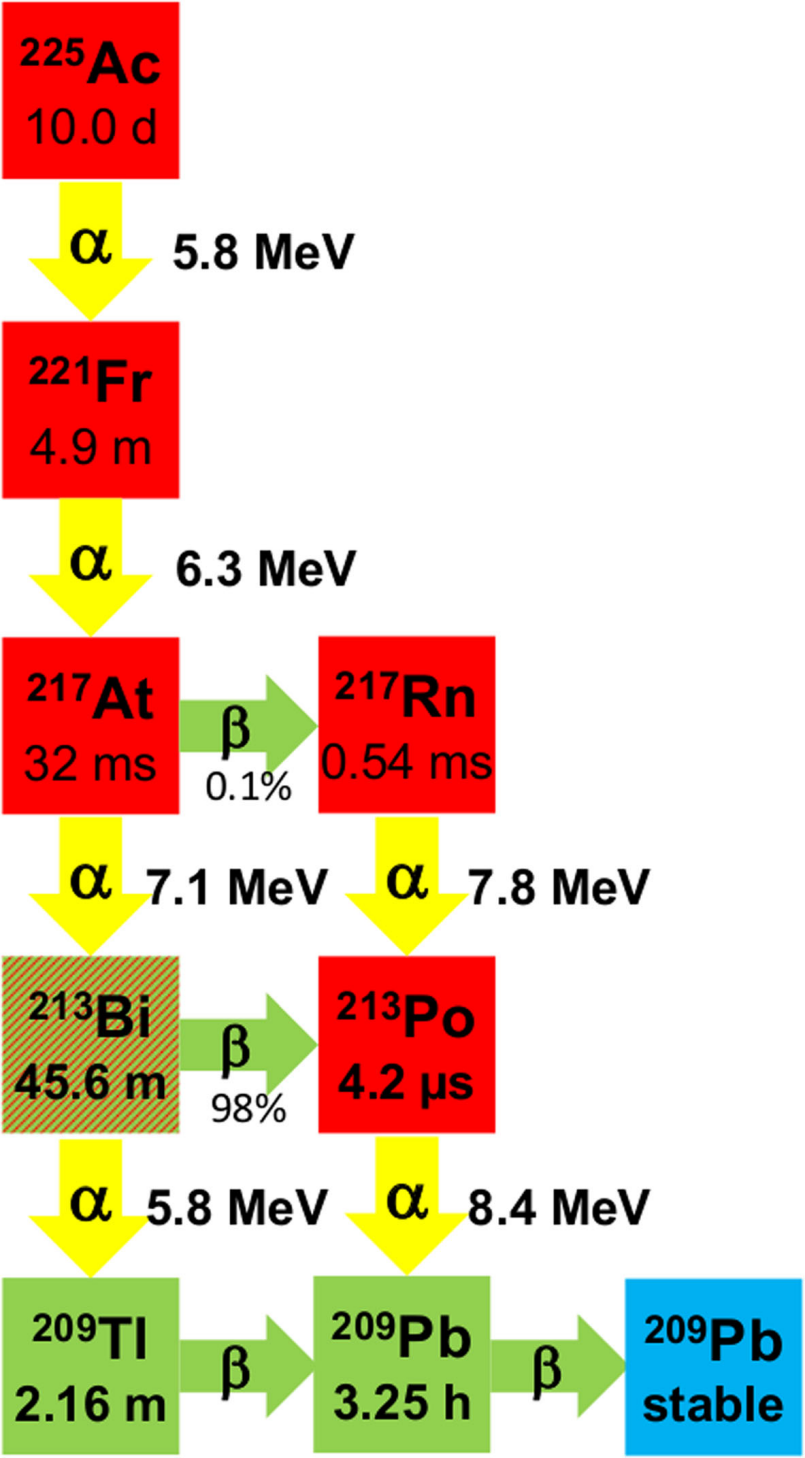
^225^Ac decay scheme (Data retrievd from J. Magill, G. Pfennig, J. Galy (2006), Karlsruher Nuklidkarte, 7th ed.; Haberbeck GmbH,Germany)

**Fig. 3:**
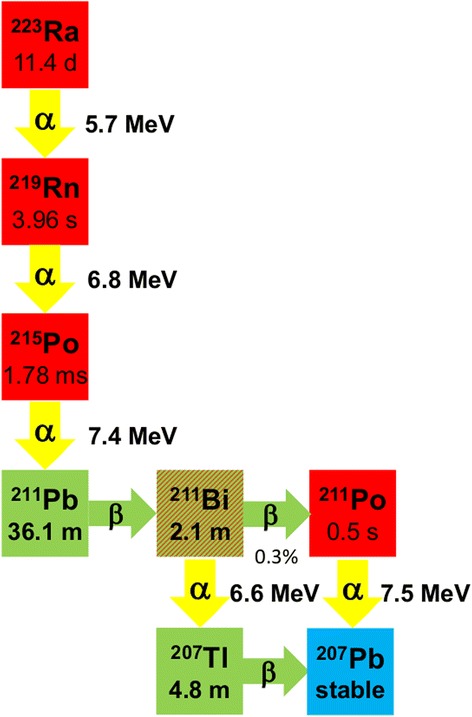
^223^Ra decay chain (Data retrievd from J. Magill, G. Pfennig, J. Galy (2006), Karlsruher Nuklidkarte, 7th ed.; Haberbeck GmbH,Germany)

**Fig. 4:**
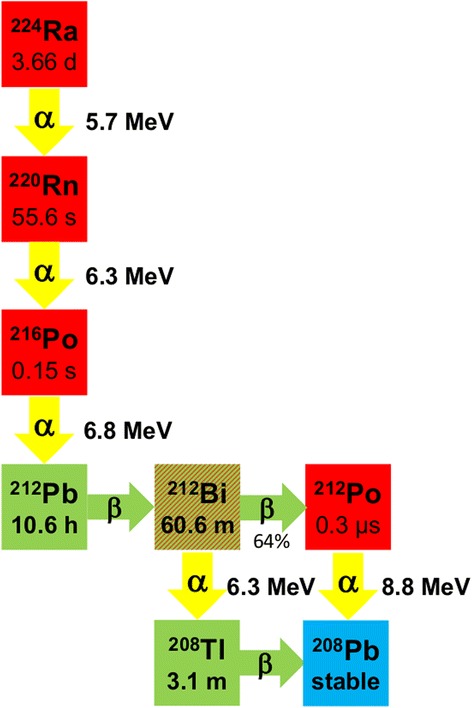
^224^Ra decay scheme(Data retrievd from J. Magill, G. Pfennig, J. Galy (2006), Karlsruher Nuklidkarte, 7th ed.; Haberbeck GmbH,Germany)

Figures 1, 2, 3 and 4 show that each decay scheme starts from an α-emitter with a half-life of several days, which would provide enough time for targeting strategies even in the case of slowly diffusing vectors. In the decay chains, along which several α-particles are emitted, the daughter radionuclides that are generated can have quite different chemical properties. For example, the first decay step of ^223^Ra and ^225^Ac leads to the formation of the noble gas ^219^Rn and the alkali metal ^221^Fr respectively, which behave chemically and physically very different from their parent nuclides. Worse than the chemical problems with the daughter radionuclides are their recoil energies that are orders of magnitudes higher than chemical bond strength. This renders chelation or chemical bonding using bifunctional ligands inefficient.

The following analysis neglects the emission of β-particles and γ-photons because they contribute only a minor fraction to the total decay energy. Moreover, this energy is distributed in a much larger volume due to the low LET and long range of β-radiation and causes less harm than the off-target radiation of released α-particle emitters.

Figure 1 shows the decay scheme of ^230^U which may be used as a generator of ^226^Th. The half-life of ^226^Th of only 31 minutes entails all problems and application restrictions that have already been mentioned for ^213^Bi. The direct use of ^230^U with a half-life of 20.8 days requires a carrier that safely retains ^226^Th and also ^222^Ra due to their half-lifes of 31 m and 38 s, respectively. The half-life of ^222^Ra is already very short, but assuming release in a post-capillary venule where the velocity of blood has its lowest values of 0.1 mm/s (Intaglietta et al., 1975), free ^222^Ra could move more than 3 mm away from the position of planned treatment and subsequently emitted α-particles would irradiate tissue outside the targeted volume. The same considerations applied to ^218^Rn (*T*_1/2_ = 38 ms ) and ^214^Po (*T*_1/2_ = 164 μs) show that the α-particles emitted by these radionuclides would still have a reasonable chance to reach the target volume. The decay product of ^214^Po is ^210^Pb, which directly emits an α-particle or undergoes a β-decay leading to the emission of a further α-particle. However, due to the very long half-life of 22.3 years the treatment is practically finished with the formation of ^210^Pb as the dose rate drops drastically. Even though the highly toxic ^210^Po (*T*_1/2_ = 138.4 d) appears in the decay scheme, it will be formed in very small amounts and will decay in radioactive equilibrium with its very long-lived mother radioisotope with an effective half-life of also 22.3 years. Whether this can be tolerated has to be assessed on the basis of detailed and careful risk-benefit analysis taking into account the totally applied activity of ^230^U in the treatment, the disease to be treated and the age of the patient. If so, the use of ^230^U offers the possibility to profit from 5 α-particles for therapy, due to the very short half-lifes of ^218^Rn and ^214^Po and the very long one of the "quasi stable" ^210^Pb, while only the first two recoiling daughter radionuclides in the decay chain must be retained by the carrier entity.

Figure 2 depicts the decay chain of ^225^Ac which has already been successfully applied in cancer therapy as outlined earlier. If a treatment cannot profit from cell internalisation a carrier has to ensure the safe confinement of ^221^Fr, ^217^At and ^213^Bi, i.e., three recoils. The ^213^Bi exhibits a half-life of 45.6 m and directly emits an α-particle (2%) or decays into ^213^Po (*T*_1/2_ = 4.2 μs), which emits the last α-particle of the decay chain. Due to the short half-life, the escape of ^213^Po from the carrier may be tolerated as it cannot move far away from the targeted treatment site. Moreover, since ^213^Po as well as ^217^Rn (formed earlier in the decay chain with a probability of 0.1%, see Fig. 2) are the products of a β-decay, their recoil energies will be very small and an escape from the carrier will be very unlikely.

Figure 3 shows the decay chain of ^223^Ra, which is already in approved clinical use for the treatment of bone metastasis resulting from prostate cancer and is well-retained in osseous tissue. The daughter nuclides in the sequence ^219^Rn and ^215^Po exhibit very short half-lifes leading to the β-emitter ^211^Pb with a half-life of 36.1 m that decays into ^211^Bi, which is either directly emitting a further α-particle or is undergoing a β-decay into ^211^Po followed by α-particle emission. Again, since the ^211^Bi and the ^211^Po are created by a β-decay their recoil energies are small and can be managed. Therefore, the last critical step is to ensure that ^211^Pb is safely confined within the nanocarrier.

When using ^224^Ra as α-particle generator the situation is similar to ^223^Ra as can be seen comparing Fig. 3 and Fig. 4. Also in this case three recoils have to be safely confined in the carrying entity until ^212^Pb is formed. As before, since the last α-particle emitters ^212^Bi and ^212^Po are formed by β-decays their recoil energies are very small.

### Recoil energies and recoil ranges

In order to determine the proper dimension of a nanocarrier, we first need to calculate the recoil energies of the daughter nuclides and in a second step their displacement range in material the nanocarrier is made of. The kinetic energy *E*_r_ of the recoiling daughter nuclides after an α-decay can be calculated as

1 $$ {E}_r=\frac{m_{\alpha }}{m_r}{E}_{\alpha, } $$

where *E*_α_ denotes the kinetic energy of the emitted α-particle and *m*_r_ and *m*_α_ denote the mass of the recoiling daughter radionuclide and the mass of the α-particle, respectively (Podgoršak, 2006). For the calculations the highest α-particle energies were used that have a meaningful intensity as reported in Table 2. For the maximum kinetic recoil energy *E*_r_(max) a daughter nucleus receives following a β-decay, the maximum energy of the β-particle *E*_β_(max) must be used and *E*_r_(max) can be calculated as

2 $$ {E}_{r\left(\max \right)}=\frac{m_e}{m_r}{E}_{\beta}\left(\max \right)\left\{1+\frac{E_{\beta}\left(\max \right)}{2{m}_e{c}^2}\right\}, $$

**Table 2:** Properties of the α-particle emissions in the decay chain of ^225^Ac: The physical half-life of the decay *T*_1/2_, the kinetic energies of the emitted α-particles *E*_α_ and of therecoiling daughter nuclides *E*_r_ are compiled

Mother radio-nuclide	T_1/2_	Daughter radio-nuclide	Energy of α-part. *E*_α_ in keV	Energy of recoiling daughter nuclide *E*_r_in keV	Range of recoiling daughter nuclides *R*_r_ in nm				
					water	amor-phous silica	graphite	zeolite	gold
^230^U	20.8 d	^226^Th	5888.4	104.2	85	46	39	42	10.6
^226^Th	31 m	^222^Ra	6336.8	114.2	89	48	38	42	11.3
^225^Ac	10 d	^221^Fr	5830.0	105.5	86	47	44	43	10.8
^221^Fr	4.9 min	^217^At	6341.0	116.9	92	50	47	43	11.7
^217^At	32 ms	^213^Bi	7066.9	132.7	101	55	52	47	12.9
^223^Ra	11.4 d	^219^Rn	5871.3	107.2	91	47	41.5	36	10.9
^219^Rn	3.96 s	^215^Po	6819.1	126.9	96.5	52	44	45.5	12.4
^215^Po	1.78 ms	^211^Pb	7386.2	140.0	104.5	57	48	49.5	13.5
^224^Ra	3.68 d	^220^Rn	5685.7	103.4	84.5	46	38	40	10.7
^220^Rn	55.6 s	^216^Po	6288.1	116.4	91.5	49.5	41.5	43	11.7
^216^Po	0.145 s	^212^Pb	6778.3	127.9	99	53.5	45	47	12.7

The α-particle energies and half-lifes *T*_1/2_ were retrievd from J. Magill, G. Pfennig, J. Galy (2006) Karlsruher Nuklidkarte, 7th ed.; Haberbeck GmbH, Germany. The range of the recoils in water, amorphous silica, graphite, zeolite and gold were determined using the simulation software SRIM (Ziegler et al., 2013)

where *m*_e_ denotes the rest mass of an electron and *c* the velocity of light (Podgoršak, 2006). Using the β-decay energies (retrieved from the International Atomic Energy Agency's Nuclear Data Services) we can calculate that for the present considerations the maximum recoil energy that needs to be considered after a β-decay is about 10 eV. Such energies are typically related with a recoil range of a few interatomic distances only. Therefore, it appears justified to neglect recoils after β-decay for the present purposes.

Based on the recoil energies we can now determine the range of the recoils in various materials that are currently considered as drug carriers in nanomedicine. For this purpose the simulation software SRIM (Ziegler et al., 2013) was used. For simplicity, when simulating the ion ranges in liposomes or polymeric nanoparticles, we replaced the carrier material by using water as stopping medium with a density of 1 g⋅cm^-3^ which is close to the density of most polymer materials. The recoil ranges determined in this way are reported in Table 2. The results show that recoil ranges of the various recoiling daughter nuclides are very similar for a given nanoparticle material, for example, in gold they are typically around 12 nm. Therefore, a nanoparticle that carries α-particle emitters in its center and is surrounded by a gold layer of 36 nm should retain all radionuclides up to the third daughter. Assuming a nucleus diameter of (3 - 5) nm housing the radioactive payload in the nanoparticle, the diameter of a gold-encapsulated α-particle nanogenerator should not exceed approximately 75 nm before surface functionalization. Similarly, when using ^230^U, gold nanoparticles with diameters as small as about 50 nm in diameter could ensure safe confinement of all daughter radionuclides up to ^222^Ra.

In Table 2 the range of the recoiling daughter isotopes are compiled in water, amorphous silica, graphite and gold. This material selection is motivated by encapsulation strategies in liposomes with aqueous content, and assuming that silica nanoparticles (Munaweera et al., 2014) or (multiwalled) carbon nanotubes (Ménard-Moyon et al., 2010) or gold nanoparticles (Vigderman and Zubarev, 2013) could be used as carriers as it is frequently suggested in literature. It should be mentioned that the much lighter α-particles lose only a tiny fraction of their energy when passing through a nanoparticle. Hence, their range in tissue is practically not affected.

### Refining the size of α-particle nanogenerators - A random walk model of recoil sequences

**Fig. 5:**
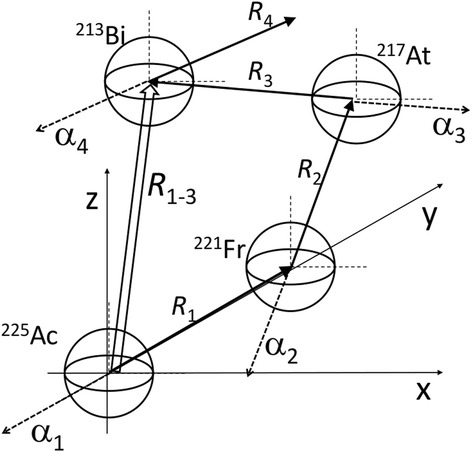
A realistic scenario of an α-particle emission cascade must take into account that the α-emissions are isotropic and statistically not correlated. ^225^Ac emits an α-particle in –γ-direction and the recoiling ^221^Fr moves the distance *R*_1_ in +γ-direction. The α-particle emitted by ^221^Fr will most likely be emitted in a different direction, hence, the recoiling daughter nucleus ^217^At recoils in a different direction by a distance *R*_2_. The subsequent α-decay will leave the ^213^Bi in a distance *R*_3_ from the decaying ^217^At. In a 3-dimensinal space the distance *R*_1-3_ between the mother nuclide ^225^Ac and the last daughter to be confined ^213^Bi is usually much smaller than *R*_1_+*R*_2_+*R*_3_≈3*R*

So far we have assumed that the required thickness of a shell would be the sum of all recoil ranges, which certainly achieves 100% recoil retention. However, α-particles are emitted isotropically and the emission directions of successive decays in the decay chains are not correlated. Therefore, the case of all α-particles being emitted in the same direction, corresponding to all recoils moving in the same direction, is highly unlikely. The distance between the position of the mother radionuclide (before undergoing the first α-decay) and the final position of the last recoil that has to be safely confined is almost always smaller than the sum of the involved recoil ranges, as illustrated in Fig. 5 for the example of ^225^Ac. The three recoils that need to be confined have all approximately the same range which simplifies the problem, thus *R≈R*_1_≈*R*_2_≈*R*_3_. The distance between the position of the mother radionuclide and the third recoil when it comes to rest can be determined by solving a random walk problem with three equally distant steps in arbitrary directions in a three-dimensional space. For a random walk problem the mean distance *r*_m_ from the starting point of the first step to the end point of the last of *N* steps is given by

3 $$ {r}_m\approx \sqrt{\frac{2}{d}N\cdot}\frac{\Gamma \left(\frac{d+1}{2}\right)}{\Gamma \left(\frac{d}{2}\right)}\cdot R $$

where *d* denotes the dimension of the problem and Γ($\frac{d+1}{2}$) and Γ($\frac{d}{2}$) represent the values of the Γ-function whose arguments become 2 and 1.5, respectively, when setting *d* = 3 for a random walk in a three-dimensional space^2^ (Dutka 1985; Johnson 1966). This gives for *r*_m_ a value of about 1.6⋅*R* which is much smaller than the worst case scenario of 3⋅*R*. However, in order to estimate the minimum nanoparticle size that maintains the recoil-retention probability above a certain predefined value, we need to know the fraction of recoil sequences that are retained in a nanoparticle with a radius *R*_NP_ smaller than 3*R*. Hence, it is necessary to calculate the probability to arrive in a 3-dimensional space after three steps with completely random orientations and equal step length *R* in a distance *r* + d*r* from the starting point. This random walk problem has been solved by Rayleigh in 1919 (Lord Rayleigh, 1919; see Appendix). For the present purposes the cumulative probability must be calculated that a certain fraction of recoils is retained within a radius *r* from the origin. It is obvious that this probability reaches 1 for *r* = 3*R*.

**Fig. 6:**
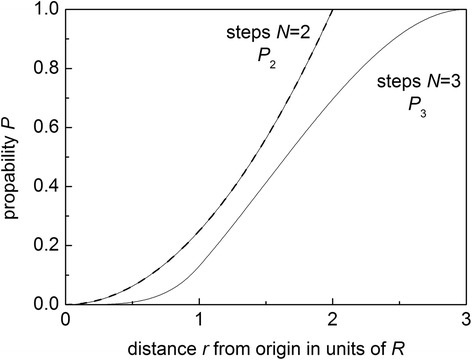
Based on the results of Rayleigh (1919, see Annex) the cumulative probability was calculated that in a 3-dimensional space a sequence of *N* = 2 and *N* = 3 random steps with equal length *R* and arbitrary orientation end in a distance *r* from the origin of the first step

The result for *N* = 2 and *N* = 3 recoil steps is visualised in Fig. 6, which shows the probability that the second (*N* = 2) and the third daughter nuclide (*N* = 3) in the decay chain come to rest within a radius *r,* which is equivalent to the statement that the 3^rd^ and the 4^th^ α-particle in the decay chain is emitted from inside this radius, respectively. Taking now the case of nanoparticles we start with the assumption that the mother radionuclide in the decay chain is located in the centre of the nanoparticle. It emits the 1^st^ α-particle, which is always emitted from inside the nanoparticle. If the nanoparticle radius is smaller than the range of the first recoiling daughter nuclide (*r*<*R*) it cannot be confined within the nanoparticle and all further α-particles would necessarily be emitted in an uncontrolled way from outside the nanoparticle. If the radius is just *r* = *R* also the 2^nd^ emitted α-particle is emitted from the inside of the nanoparticle. Additionally, the 2^nd^ recoiling daughter has a chance to move in a direction towards the inside of the nanoparticle and to be stopped inside. This implies that also the 3^rd^ emitted α-particle has a certain probability for being emitted from inside the nanoparticle, with this probability reaching 1 when the nanoparticle radius reaches *r* = 2*R*. From Fig. 6 it can be recognized that the 3^rd^ recoil can be stopped in a bulk material in a distance *r*<*R*, but such small nanoparticles would already have released the first daughter in the decay chain, thus subsequent recoil to *r*<*R* is excluded. Therefore, for nanoparticles the probability has to be set to zero for *r*<*R*. In the range *R*<*r*< 2*R* the probability to confine the third recoiling daughter radionuclide must be corrected for those cases in which the second daughter radionuclide was already ejected out of the nanoparticle. This correction becomes zero for *r* = 2*R*. From this radius on, the curve for *N* = 3 steps in Fig. 6 describes properly the retention probability of the third recoiling daughter nuclide in the nanoparticle with more than three α-particles being emitted from inside the nanoparticle.

**Fig. 7:**
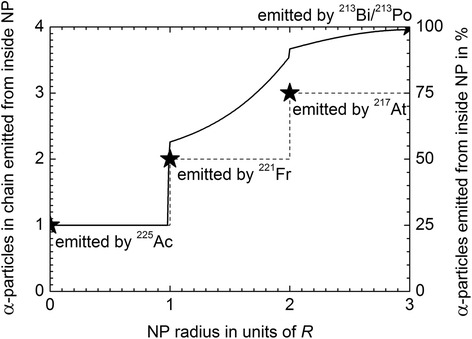
The left y-axis denotes the number of α-particles in the decay chain of ^225^Ac that are emitted from inside a nanoparticle with radius *r* measured in units of the recoil range *R* of the decay products of ^225^Ac (i.e., of ^221^Fr, ^217^At and ^213^Bi), which is very similar for a given material. It is assumed that the ^225^Ac is located in the centre of the nanoparticle at *r* = 0. The step function refers to the worst case that all recoils are lined up in the same direction. Setting the similar recoil ranges of ^221^Fr, ^217^At and ^213^Bi to *R*, these daughters of ^225^Ac are fully retained in nanoparticles with a radius *R*, 2*R* and 3*R*, respectively. The more realistic model considering the sequence of recoils as random steps in arbitrary directions in the three-dimensional space yields a higher recoil retention for nanoparticles larger than *R*. The right y-axis translates recoil retention into the fraction of α-particles that are emitted from the inside of the nanoparticle

Figure 7 depicts the splined solutions of the preceding considerations in order to assess how many of the 4 α-particles emitted in the decay chain of ^225^Ac are emitted from inside the nanoparticle as a function of its radius. The deviation from the step function, that increases by 1 whenever a multiple integer of *R* is reached, is due to the completely random orientations between the α-particle emissions in the decay chain that can be calculated using a random walk model. Figure 7 may be adopted for the cases of the decay chains of ^223^Ra and ^224^Ra, in which three recoiling daughter nuclides have to be controlled. Since for a given material the recoil ranges of all daughters in the decay cascade are similar, the nanoparticle size in Fig. 7 is normalised to the recoil range *R*. Thus, for a given carrier material and mother radionuclide a typical recoil range can be estimated from Table 2. Using this recoil range as unit value, from Fig. 7 the radius of the nanoparticle can be derived that retains a certain fraction of recoils. From the retention of recoils the fraction of α-particles emitted in the decay cascade from inside the nanoparticles can be derived in a straightforward manner and is depicted on the right y-axis in Fig. 7. However, it should be noted however that for these calculations to be correct the mother radionuclides of the decay chains must be localised at the centre of the spherical nanoparticles.

## Discussion

Small nanoparticles have usually a better biodistribution and faster clearance than bigger ones, but when loaded with α-particle emitters at the expense of lower recoil retention. Surprisingly high recoil retention data have already been reported for certain approaches *in vitro* that were sometimes explained by re-adsorption of the decay products by the carrier. However, *in vivo* re-adsorption is highly unlikely due to adsorption to abundantly available blood proteins and due to immediate separation of released daughter nuclides from their carrier in the blood stream (de Kruijff et al., 2015). When striving for small nanoparticles that can nevertheless safely confine all recoiling daughters that occur in an α-particle cascade, the strategy suggested by Woodward et al. (2011) and McLaughlin et al. (2013) using small core nanoparticles that are loaded with the α-emitters and then surrounded by confining shells preferentially with high-Z materials appears to be the most promising and most universal approach. However, it appears still difficult to predict the adequate thickness of the surrounding shell.

McLaughlin et al. (2013) state a *de facto* quantitative retention of ^225^Ac and of about 90% of ^221^Fr after 3 weeks in [La,Gd]PO_4_ nanoparticles with a diameter of 27 nm. In an earlier work, Woodward et al. (2011) state about 40% retention of ^221^Fr in [^225^Ac]LaPO_4_ nanoparticles with a diameter of 3-5 nm after 25 days. The range of recoiling of ^221^Fr in LaPO_4_ and GdPO_4_ is about 20 nm. Thus, in both cases one would not expect such a high retention of ^221^Fr. In the case of the small 3-5 nm sized nanoparticles the authors suggest that a part of the recoil energy may be transferred to the whole entity of the nanoparticle (Woodward et al., 2011). This is however highly unlikely since a 5 nm nanoparticle, whose weight was specified by the authors with *M*_2_ = 200 kDa, would be much heavier than a ^221^Fr atom with *M*_1_ = 221 Da, which renders the energy fraction transferred to the nanoparticle approximately *M*_1_/(*M*_1_+*M*_2_) ≈ 10^-3^. The authors did not describe the mechanisms of such a transfer in detail. However, the simulation software SRIM (Ziegler et al., 2013) takes already into account all conceivable types of interactions between energetic ions and the host material in which they are slowed down, as for example head-on collisions of the recoils with atoms of the host material leading to collision cascades and atomic displacements of secondary ions, electronic and phononic excitations. Finally, all processes by which ions lose kinetic energy in bulk matter lead to the production of heat and defects in the crystal lattice. Therefore, it will be difficult to hypothesize a process that allows a partial transfer of momentum and energy from the recoiling daughter radionuclide to the entity of a nanoparticle. A much simpler explanation for the higher than expected retention could be based on nanoparticle agglomeration, where the ^221^Fr that escapes one nanoparticle is implanted in an adjacent one and finally retained there. Radiolabelling of nanoparticles by recoil implantation of radioactive atoms has been applied routinely by one of the present authors (e.g. Holzwarth et al., 2014). Recoil implantation may even explain the 40% of ^221^Fr retention in perfectly dispersed 3-5 nm sized [^225^Ac]LaPO_4_ nanoparticles. With the information given by Woodward et al. (2011) (600 μCi ^225^Ac, one ^225^Ac atom in 30 nanoparticles and dispersion volume 0.5 mL) it is possible to estimate the number and mean distance of the nanoparticles used for this leaching experiment. One obtains a mean distance of the nanoparticles of about 85 nm which equals the range of the 105 keV ^221^Fr in water. A detailed SRIM simulation assuming ^221^Fr is emitted from the centre of a 4 nm sized LaPO_4_ nanoparticle and passing 85 nm in water gives a residual kinetic energy slightly higher than 1 keV, which is sufficient to implant it again in a depth of 2 nm below a LaPO_4_ surface. Thus, using a slightly lower concentration might have shown the expected non-retention of ^221^Fr in so small nanoparticles or would have supported the more likely hypothesis of a certain degree of nanoparticle agglomeration.

The approach to surround a small radioactively loaded nanoparticle with recoil-confining shells might pave the way for a broader use of Ra-nuclides in targeted α-particle therapy. If a useful quantity of Ra could be loaded on very small nanozeolites (Piotrowska et al., 2017; 2013) and if these can be coated with a biocompatible material of sufficiently high density and therefore higher stopping power for energetic ions, the discussion of whether or not re-absorption of recoiling daughters onto the surface of nanozeolites could explain their low release rates and whether this might be reproduced *in vivo* would become obsolete. The reported retention of more than 90% of the daughter nuclides in nanozeolites with a TEM-derived diameter of 60 nm (Piotrowska et al., 2017) is by far higher than expected considering the random walk model since a radius of only 30 nm would even be smaller than the recoil range of ^219^Rn. However, when using the hydrodynamic diameter determined by DLS of around 160 nm, more than 85% recoil retention could be expected. Nevertheless, the loading conditions of the nanozeolites by synthesis in presence of the radionuclides or by surface adsorption are very different from the model presented here that assumes the radioactive load being located in the centre of a spherical nanoparticle.

Using liposomes or polymersomes as carriers has the disadvantage that the densities are too close to 1 g⋅cm^-3^, hence, they exhibit a small stopping power for ions being equivalent with long ion ranges requiring large structures to minimise the release of recoiling daughters into the environment. As long as no mechanism is provided that keeps the mother nuclide in the centre of the nanocarrier, one must assume that all retained radionuclides occurring in the decay chain are homogeneously distributed within the liposomes. Considering a recoil range of typically 100 nm in water as indicated in Table 2, a liposome with a diameter of 650 nm homogeneously loaded with ^225^Ac, as used by Sofou et al. (2004), will safely retain ^221^Fr only in an inner volume of 450 nm in diameter, which accounts for about 1/3 of the total volume. Thus 2/3 of the created ^221^Fr are in a distance of less than 100 nm from the surface. Assuming that half of it recoils towards the inside and half towards the outside of the liposome, a total of 2/3 are retained. Assuming the same for the decay products ^217^At and ^213^Bi, the retention of ^213^Bi is (2/3)^3^≈ 30%. Such a value was reported by Sofou et al. (2007) using multivesicular liposomes, but even much lower values were reported (see Table 2). However, whether an even lower retention rate may be tolerated for much smaller liposomes depends on the advantage that such a carrier could provide concerning rapid penetration and extravasation into tumour tissue and whether the carrier is rapidly internalised by the target cells or not.

In summary, carrier size is an important parameter but the best value to ensure maximum recoil retention is not necessarily the best value when optimising for therapeutic success since various parameters have to be balanced (McDevitt et al., 2001; Kim and Brechbiel, 2012). Important related aspects are the ease and flexibility of surface functionalisation in order to achieve stabilisation in physiological conditions as well as fast, high and persistent uptake in the target tissue. The use of radionuclides with a half-life of more than one week requires a sufficiently persistent retention in the target tissue for several half-lifes, ideally until complete decay is achieved. Radionuclides leaking out of the target tissue, whether individually or as nanoparticulate may cause off-target toxicity. Especially nanoparticles that may be recognized and stored in cells of the reticuloendothelial system, will deliver a large dose to organs of the reticuloendothelial system. In such cases biodegradable nanoparticles would be of advantage that release the radionuclides individually and would allow for accelerated excretion by additionally applying metal scavengers and diuretics (Jaggi et al., 2005) possibly supported by other measures to protect critical organs (cf. de Kruiff et al., 2015). The excretion of complete nanoparticles will most likely be slow as they will not pass renal clearance and may only be excreted following slow hepatobiliary pathways (Kreyling et al., 2017). However, in this context the approach of de Kruijff et al. (2017) could offer the possibility to achieve targeting with biodegradable polymeric nanocarriers which contain their radioactive load incorporated in a small nanoparticle inside the carrier. Provided this radioactive 'inner' nanoparticle is small enough to pass renal clearance, untargeted radiation might be excreted sufficiently fast before causing harm after degradation of the outer polymeric carrier shell. However, many physiological details need to be considered and mastered to make such a sophisticated approach a success.

Furthermore, the encapsulation of α-particle emitters that originate an α-particle cascade may necessitate to consider nanoparticle degradation aspects that are not relevant for any other application. Recoiling atoms with kinetic energies as high as (100 – 200) keV may have similar effects on small nanoparticles as those observed when nanoparticles are externally bombarded with heavy ions leading to material loss by ballistic and/or evaporative sputtering (Greaves et al., 2013; Järvi and Nordlund, 2012; Zimmermann and Urbassek, 2008). The formation of so-called thermal spikes close to the nanoparticle surface may cause the quasi-explosive ejection of atom clusters from the nanoparticle material (Greaves et al., 2013). Such processes may locally affect the stability of chemical bonds and impair homogeneous surface functionalisation. Additionally, the dissipation of the recoil energy in a small nanoparticle will lead to a temperature increase for some 100 ns which might be long enough for diffusion processes to take place displacing encapsulated radionuclides. None of the investigations compiled in Table 1 has so far provided any indication for the relevance of such processes. However, one should be aware that such processes do exist and they might show up when unfavourable combinations of carrier size, carrier material and loaded activity are encountered.

In spite of these latent concerns, the possibility to use longer-lived mother nuclides as *in vivo* α-particle nanogenerators in cancer therapy can provide significant advantages. The pros and cons of directly using ^225^Ac (*T*_1/2_ = 10 d) as therapeutic radionuclide instead of ^213^Bi (*T*_1/2_ = 43.6 min), which is usually eluted from a ^225^Ac/^213^Bi generator, have been investigated by Allen (2017). The total energy released by four α-decays of 27.6 MeV provides a much higher irradiation dose in the same volume than the 8.4 MeV released by the α-decay of ^213^Bi alone. Thus, using ^225^Ac the same dose could be administered with a fraction of the applied ^213^Bi activity, thereby increasing therapeutic efficiency and making a much more economic use of ^225^Ac, which would otherwise release the energy of three α-particles of 19.2 MeV uselessly in the columns of an ^225^Ac/^213^Bi generator. Allen (2017) concluded, that when normalised to equal α-production, ^225^Ac has a higher therapeutic gain than ^213^Bi, where the therapeutic gain is defined as the cell survival of non-targeted cells divided by the survival of targeted cancer cells. Allen (2017) also showed that ^225^Ac is much more toxic for targeted cancer cells than ^213^Bi, while this is not the case for non-targeted cells. Taking into account dose rate and repair mechanisms for double strand breaks, Allen (2017) concludes that ^225^Ac is performing better or equal to ^213^Bi at a much lower cost. Similar results may be expected for the generator systems ^230^U/^210^Pb, ^223^Ra/^211^Pb and ^224^Ra/^212^Pb (cf. Figs. 1, 2, 3 and 4).

The random walk model presented here estimates the retention of daughter nuclides in spherical nanoparticles and the fraction of α-particles emitted from inside the nanoparticle as a function of its size. The experimental verification of this model requires the availability of stable, non-aggregated, colloidal nanoparticle suspensions. In any case, for medical applications agglomeration problems need to be solved, since excessive size seriously compromises the biodistribution and the targeting capabilities of the constructs, and aggregation of colloidal nanoparticles in physiological conditions may yield adverse outcomes.

Despite progress in nanosciences the basic problem in medical therapy remains the challenge of targeting, penetration and extravasation of the carrier into tumour tissue as well as the stability and metabolic fate of the carriers (Kim and Brechbiel, 2012; Ruenraroengsak et al., 2010).

## Conclusion

Promising clinical results using ^223^Ra and ^225^Ac in targeted radionuclide therapy justify all the efforts to develop *in vivo* α-particle nanogenerators and to enable a wider application of ^230^U, ^225^Ac, ^224^Ra and ^223^Ra. The random walk model for recoiling daughter nuclides in α-particle cascades provides an estimate for the size of an idealised spherical nanoparticle assuming that the radionuclide is localized in a very small core nanoparticle surrounded by a concentric shell structure that confines the recoiling α-emitting daughter radionuclides. Nanoparticle agglomeration is a main obstacle for the experimental verification of the model as well as for efficient tumour targeting. However, when optimising nanoparticles for targeted therapy their size is only one parameter that needs to be considered together with the ease of surface functionalisation, the time required for accumulation in tumour tissue, and the interaction with tumour cells. Fast tumour accumulation and rapid internalisation of the carrier by tumour cells may justify the use of smaller nanocarriers compromising the retention of daughter nuclides in the α-particle emission cascade. The presented random walk model could be used to estimate how much non-targeted α-particle activity may be expected when reducing nanoparticle size.

## Appendix

For a random flight with two steps of length *l*_1_ and *l*_2_ Rayleigh (1919) gets for the probability to find the end point of the flight in a distance *r* + d*r* from the origin (Eqn (56) in Rayleigh, 1919)

$$ \frac{dP_2}{dr}=\frac{r}{2{l}_1{l}_2}=\frac{r}{2{R}^2} $$

where we approximate *l*_1_ = *l*_2_ = *R* considering all recoil ranges as approximately equidistant random walk steps. For a random flight with three steps of equal length *l* = *R* the probability to find the end point of the flight in a distance *r* + d*r* from the origin (Eqn (61) in Rayleigh, 1919) is given by a solution that distinguishes three ranges

$$ r<l,\frac{dP_3}{dr}=\frac{r^2}{2{l}^3}=\frac{r^2}{2{R}^3}, $$

$$ 3l>r>l,\frac{dP_3}{dr}=\frac{3 lr-{r}^2}{4{l}^3}=\frac{3 Rr-{r}^2}{4{R}^3}, $$

$$ r>3l,\frac{dP_3}{dr}=0. $$

For the present purpose the cumulative probabilities *P*_2_ and *P*_3_ are required that a random flight ends within a distance *r*. This corresponds to the integration and normalisation of the probabilities d*P*_2_/d*r* and d*P*_3_/d*r* whose results are presented in Fig. 6.

## Footnotes

1 Before known as Alpharadin® (^223^RaCl_2_) was developed by Algeta ASA (Norway) with Bayer Schering Pharma AG as partner.

2 In this practical form the equation is only presented on the internet (Mathematics Stack Exchange, 2016) and it is an asymptotic approximation that becomes exact only for large *N* when $\left\langle {r}^2\right\rangle$ and ${r}_m\approx \sqrt{\left\langle {r}^2\right\rangle }$ follow a *χ*^2^ or *χ* distribution, respectively (see also Dutka 1985; Johnson 1966).
